# Development and testing of a new measure of social connection for long‐term care homes: the SONNET study

**DOI:** 10.1002/alz70858_098452

**Published:** 2025-12-25

**Authors:** Jennifer Bethell, Steven Stewart, Hannah Chapman, Neha Dewan, Gill Livingston, Katherine S McGilton, Sube Banerjee, Kirsten Corazzini, David Edvardsson, Madalena Liougas, Hannah M O'Rourke, Andrew Sommerlad

**Affiliations:** ^1^ University of Toronto, Toronto, ON, Canada; ^2^ KITE Research Institute, Toronto Rehabilitation Institute ‐ University Health Network, Toronto, ON, Canada; ^3^ Queen Mary University of London, London, United Kingdom; ^4^ Western University of Health Sciences, Lebanon, OR, USA; ^5^ University College London, London, London, United Kingdom; ^6^ University of Nottingham, Nottingham, United Kingdom; ^7^ University of New Hampshire, Durham, NH, USA; ^8^ School of Nursing & Midwifery, College of Science, Health and Engineering, La Trobe University, Heidelberg, VIC, Australia; ^9^ Department of Nursing, Umeaa University, Umeaa, Sweden; ^10^ University of Alberta, Edmonton, AB, Canada

## Abstract

**Background:**

Social connection comprises distinct but related aspects of human social relationships. Positive aspects of social connection are associated with better health and, in long‐term care (LTC) homes, represent a key component of quality of life, quality of care and sense of home. Despite its importance, research and reporting on social connection in this setting is limited by a lack of good quality instruments with which to measure it. Our objective was to develop and test a measure of social connection for LTC homes.

**Method:**

We conducted this study in Canada and the UK. We developed a conceptual model based on research literature and existing measures. We conducted and thematically analysed qualitative interviews with residents, families and staff to identify important aspects of social connection. We created and refined a list of candidate items that we piloted and field‐tested with LTC residents and staff. We examined descriptive statistics (e.g., missing data), dimensionality and internal consistency to further refine the measure. We evaluated the final resident (self‐report) and staff (proxy‐report) measures’ feasibility, acceptability, reliability and validity. We worked with patient and public involvement (PPI) partners in developing our methods and the Social Connection in LTC homes (SONNET) measure.

**Result:**

We prioritized social engagement and social connectedness (loneliness) from our conceptual model of social connection, based on priorities identified from qualitative interviews (*n* = 67) and PPI partners. We developed 58 candidate items for self‐report and proxy‐report which we then reduced to 20 items based on feedback from academic experts and PPI partners and pilot testing (*n* = 9). We further reduced this to 12 items based on field‐testing (*n* = 111 resident‐staff dyads) results. We tested the measurement properties with 52 resident‐staff dyads, including 33 (65%) residents with dementia. Findings supported the hypothesised two factor structure; that the SONNET scale correlated with related constructs; and good/ acceptable and reliability (internal consistency, test‐retest and inter‐rater).

**Conclusion:**

The SONNET scale assesses social engagement and social connectedness (loneliness) for LTC home residents, with resident and staff‐reported versions. It is feasible and acceptable to LTC residents and staff with promising reliability and validity, although we recommend further testing.

